# Single Tooth Replacement Using Immediately Loaded Basal Implant-Supported Fixed Prosthesis in a Hyperdense Lesion: A Case Report

**DOI:** 10.7759/cureus.34946

**Published:** 2023-02-13

**Authors:** Safiya Hassan, Prasad Dhadse, Bhushan P Mundada, Pavan Bajaj, Chitrika Subhadarsanee, Ranu R Oza

**Affiliations:** 1 Department of Periodontics and Implantology, Sharad Pawar Dental College and Hospital, Datta Meghe Institiute of Higher Education and Research, Wardha, IND; 2 Department of Periodontics and Implantology, Sharad Pawar Dental College and Hospital, Datta Meghe Institute of Higher Education and Research, Wardha, IND; 3 Department of Oral and Maxillofacial Surgery, Sharad Pawar Dental College and Hospital, Datta Meghe Institute of Higher Education and Research, Wardha, IND

**Keywords:** osseointegration, hyperdense lesions, periapical infections, condensing osteitis, basal implant

## Abstract

Osseous modifications in the periapical areas are related to chronic endodontic infections. Often, teeth with periapical infections and hopeless prognosis are removed and replaced with dental implants. In this clinical report, a patient with a radiopaque lesion on the root apex of the mandibular right first molar root is presented. Bone- and tissue-borne lesions were the differential diagnoses for the radiopaque mass. Based on the clinical and radiological characteristics, condensing osteitis (CO) was the final diagnosis of osseous growth (bone density and trabeculation of the bone). Under local anesthesia, tooth 46 was atraumatically extracted, and the immediate basal implant was placed. This case report investigated the effectiveness and safety of dental implantation in the vicinity of hyperdense lesions.

## Introduction

The placement of dental implants is widely practiced in implant dentistry. However, there are circumstances when conventional implant placement is not possible. An adequate bone must be present for trouble-free and effective implant placement (at least 13-15 mm length and 5-7 mm width). There are procedures when implant placement has to be combined with additional surgical therapies like heavy grafting procedures, direct or indirect sinus lift procedures, and nerve lateralization. These procedures are technique-sensitive and are only sometimes feasible. New treatment modalities, including short and basal implants, have emerged to overcome all the shortcomings. Basal implants are dental implants uniquely designed to gain anchorage from the basal cortical bone. In corticobasal implantology, osseointegration on or under the first cortex is neither crucial nor necessary for the functioning of the bone-implant prosthetic system [1]. When multiple teeth are missing or must be extracted after a two-stage implant placement procedure or bone augmentation has failed, or in cases of all types of bone atrophy, such as those involving very thin ridges, insufficient buccolingual thickness, or insufficient bone height, basal implants can be used [1].

Condensing osteitis (CO) is characterized by periodic bone growth. A mild dental pulp infection is responsible for bone overgrowth. CO is caused by mild chronic irritation of the root canal. Dental pulp inflammation in chronic pulpitis or low-virulence bacteria in the remaining necrotic pulp following ineffective endodontic treatment might cause bone reaction [2-4]. Localized jaw sclerosis risk factors include stress, infection, or trauma [5]. Apical bone growth occurs due to chronic pulpal inflammation. A low-grade inflammatory stimulus emerging from the pulp that induces the proliferation of osteoblasts is associated with the etiology of CO [6,7]. Histologically, CO is characterized by impaired bone remodeling, de novo bone formation, and regular bone marrow exchange with fibrous connective tissue with inflammatory cell infiltrate [8]. On radiographs, CO is seen as a well-defined, uniform, dense radiopaque mass at the apex of the tooth’s root, along with the widening of the periodontal ligament space and loss of the lamina dura [9,10]. CO can be differentiated from idiopathic sclerosis regarding its relationship with the pathological lesions of the dental pulp [11,12]. We hereby report a case of basal implant placement in CO, taking advantage of the hyperdense lesion and utilizing it as an osseofixating area. To the best of our knowledge, this is the first case report depicting the placement of the basal implant (King of Compression (KOC) Plus; Simpladent, Ghaziabad, India) in CO.

## Case presentation

**Figure 1:**
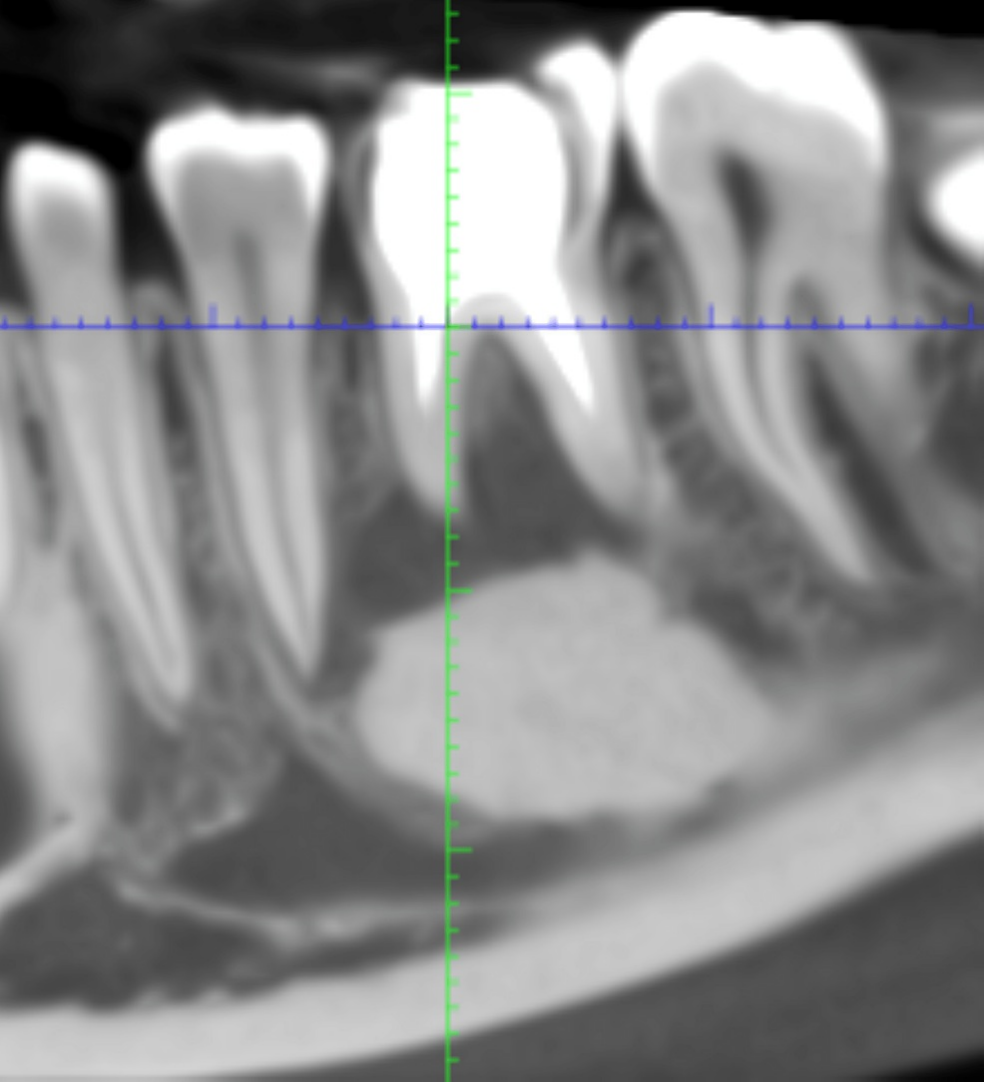
CBCT showing a well-defined dense radiopaque mass concerning the roots of 46. CBCT: cone beam computed tomography.

An 18-year-old male patient came to the Department of Periodontics and Oral Implantology with a complaint of black discoloration of the tooth’s lower right back region of the jaw for three years and gave a history of root canal treatment with 46, four years before. The patient had no systemic diseases like diabetes, hypertension, thyroid, or asthma. No history of parafunctional habits or smoking/alcohol consumption. The patient had no known drug allergies and brushed his teeth twice a day. Clinical examination revealed the presence of secondary caries with 46. No mobility was associated with the tooth, and there was no tenderness on percussion. The patient was then advised to undergo cone-beam computed tomography (CBCT). CBCT with 46 shows a well-defined dense radiopaque mass concerning the roots of 46 of size 13.82 mm × 9.91 mm, as depicted in Figure 1.

There is a loss of lamina dura and a partial loss of buccal plate, exposing the mesial root of 46. Our differential diagnoses were CO, osteosclerosis, and cementoblastoma. The tooth had been root canal treated and had asymptomatic persistent apical periodontitis. Hence, the final diagnosis given was CO with 46. The patient demanded extraction with 46 and replacement with a fixed prosthesis.

Treatment plan

Considering the patient’s age, we decided to replace it with an implant prosthesis. The procedure was explained to the patient, and informed consent was taken. However, immediate placement of a conventional implant was not possible in this case because of the presence of radiopaque mass representing CO. A significant amount of bone loss was also present, and guided bone regeneration (GBR) was indicated, followed by implant placement after five to six months. But the patient demanded an immediate replacement. Considering the significant amount of bone atrophy in 46 regions and the reduced height of bone, we decided to replace it with a basal implant. KOC Plus type of basal implant is placed.

Rationale behind the use of basal implant

Basal bone is heavily corticated; it rarely resorbs or gets infected. The crestal bone has a higher resorption rate because it is less thick and more susceptible to infections brought on by traumas, iatrogenic causes, or tooth-borne illnesses [13]. This justification is based on the finding in orthopedic surgery that cortical areas are important because they resist resorption. Orthopedic implants are another name for basal implants. Single-piece implants made of titanium-molybdenum or titanium-aluminum-vanadium alloy are known as KOC Plus implants. They comprise compression and bicortical screws. Bicortical screws are present at the apex of the implant, which osseofixates the implant. In the case mentioned above, we fixated the implant into CO as it provided dense bone in a particular area [14]. 

Procedure

**Figure 2:**
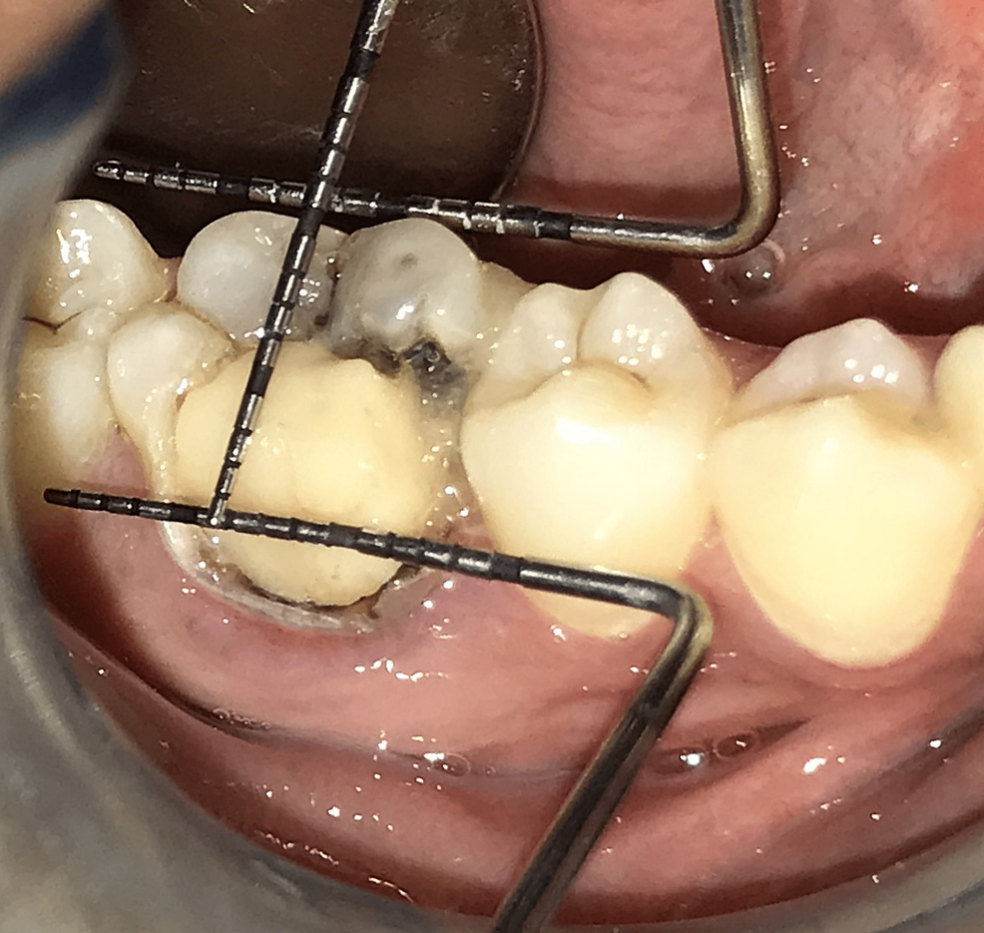
Baseline view image shows a preoperative view of 46.

The patient read and signed an informed consent form. The prophylactic antibiotic (500 g amoxicillin) was given one hour preoperatively. Local anesthesia was administered. Figure 2 depicts a preoperative view of 46. 

**Figure 3:**
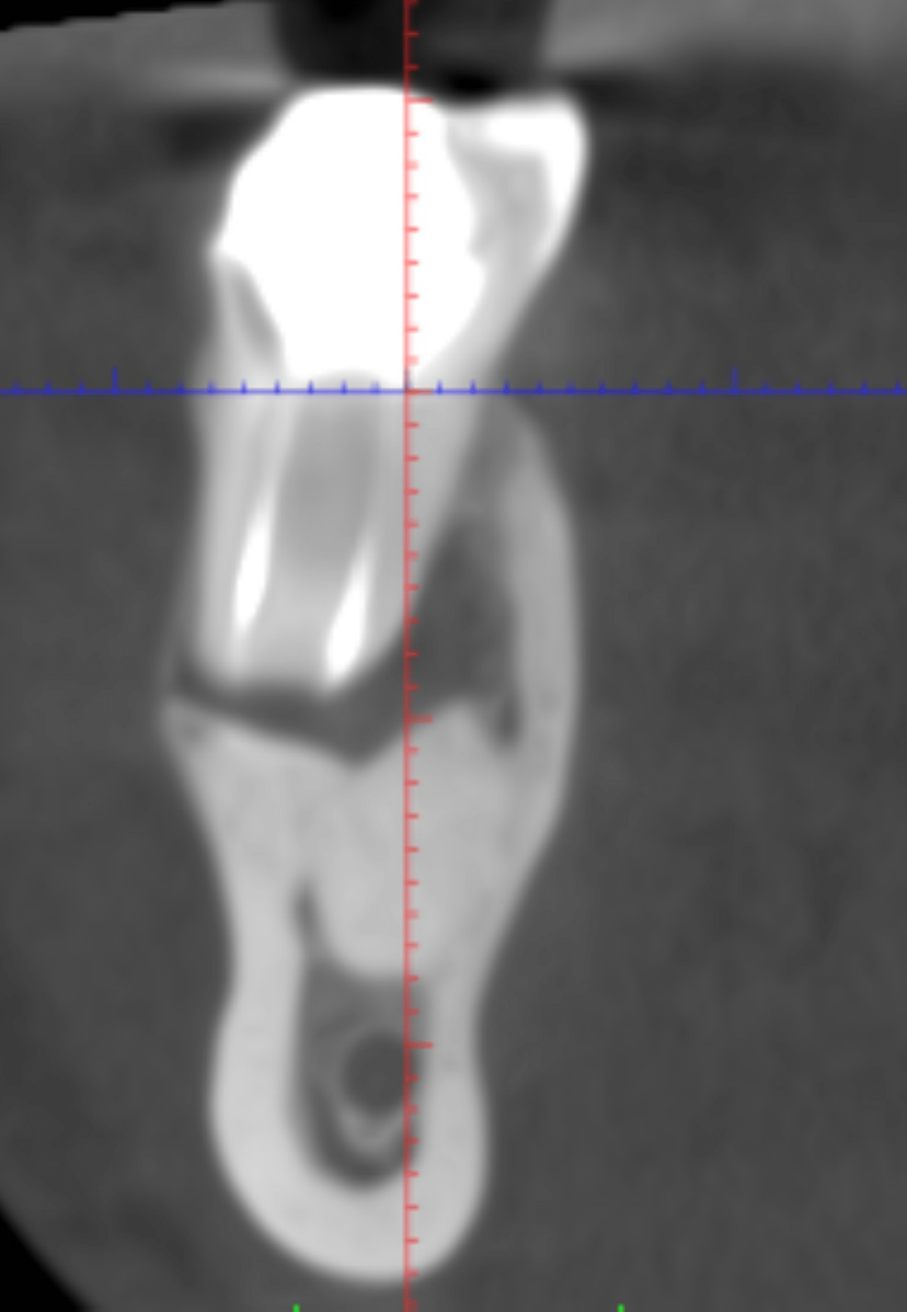
Preoperative view of CBCT with 46. CBCT: cone beam computed tomography.

Figure 3 depicts a preoperative CBCT.

**Figure 4:**
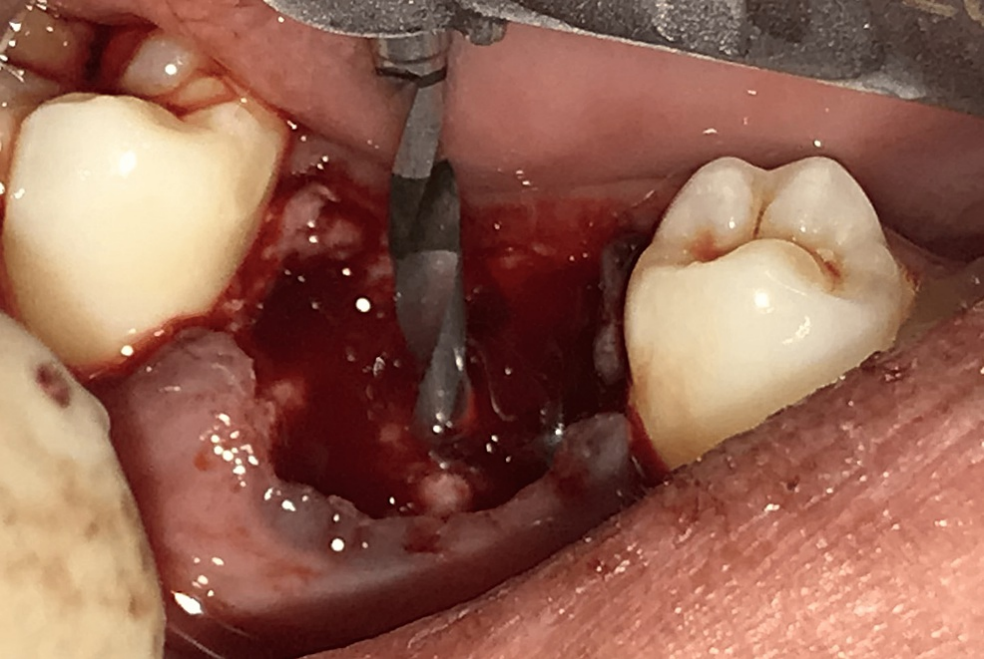
The placement of DOS 1 drill.

A sulcular incision was given, and a full-thickness mucoperiosteal flap was reflected. Periotomes were used for atraumatic extraction. Throughout the surgery, the mode of irrigation was used externally. Figure 4 depicts a single pilot osteotomy with a pathfinder Drill.

**Figure 5:**
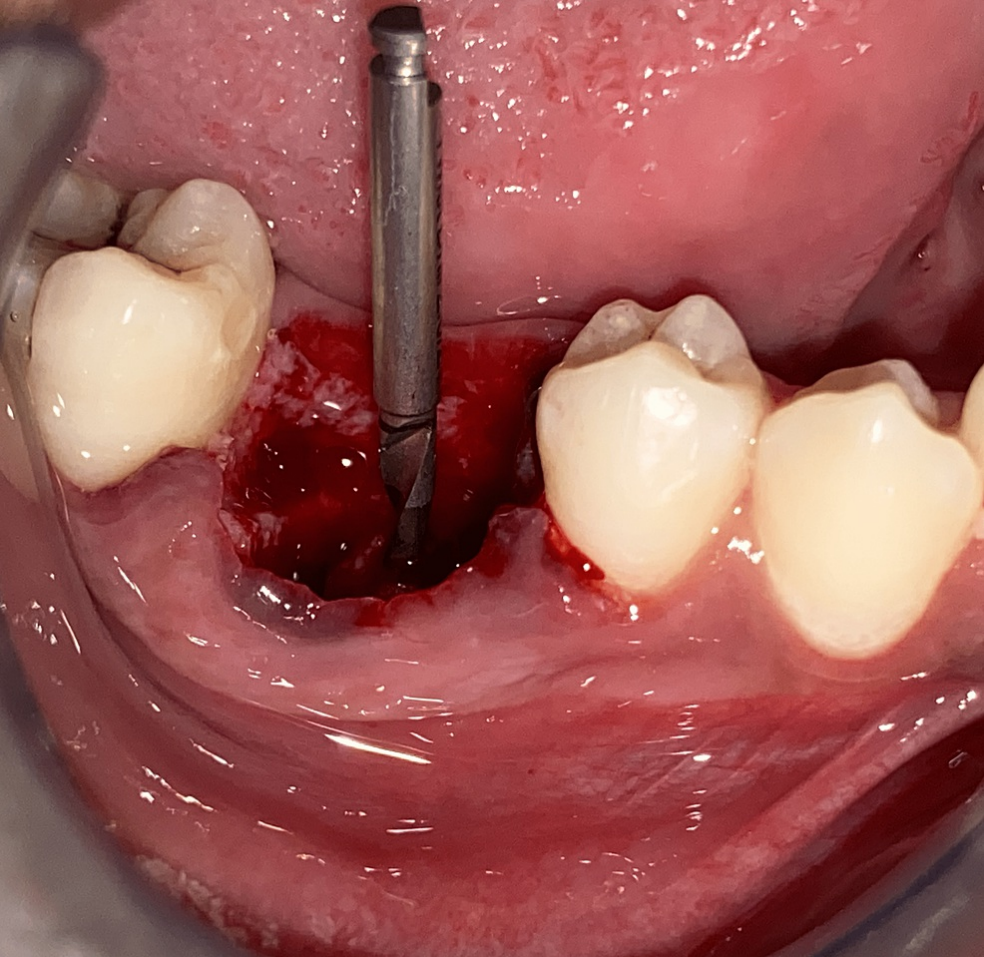
DOS 1 drill to check the angulation.

Figure 5 depicts the angulation of implant placement.

**Figure 6:**
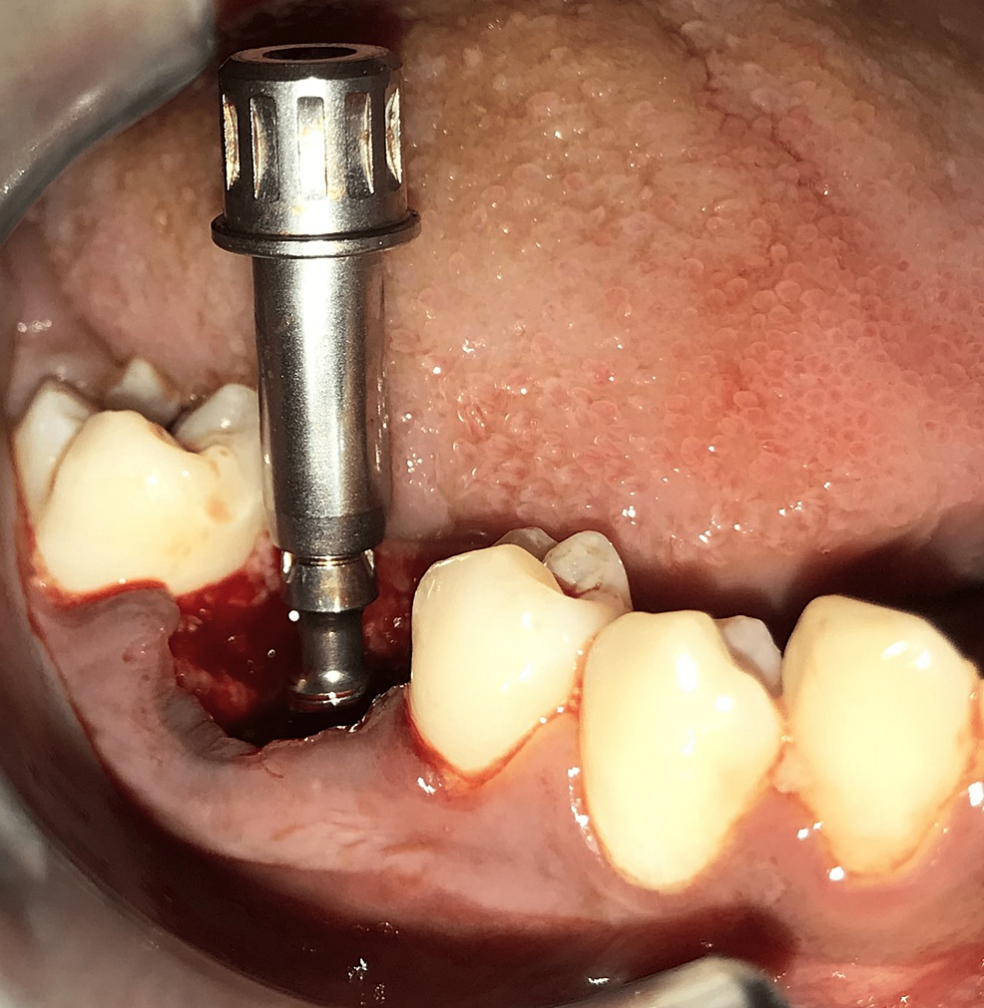
Small head insertion tool to place the implant in the implant bed site.

Sequential drilling by DOS1, DOS2, and DOS3 drills followed it. Implant bed preparation was then done. Once the implant bed preparation was done, the implant was carefully removed. Figure 6 shows a small head insertion tool.

**Figure 7:**
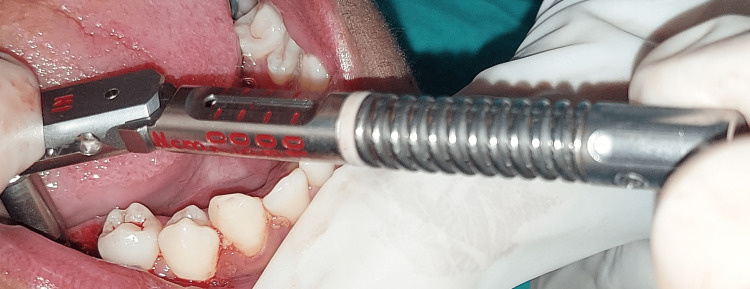
Torque wrench showing torque of 80 N/cm.

The implant was then inserted into the implant bed site. Figure 7 shows a torque of 80 N/cm.

**Figure 8:**
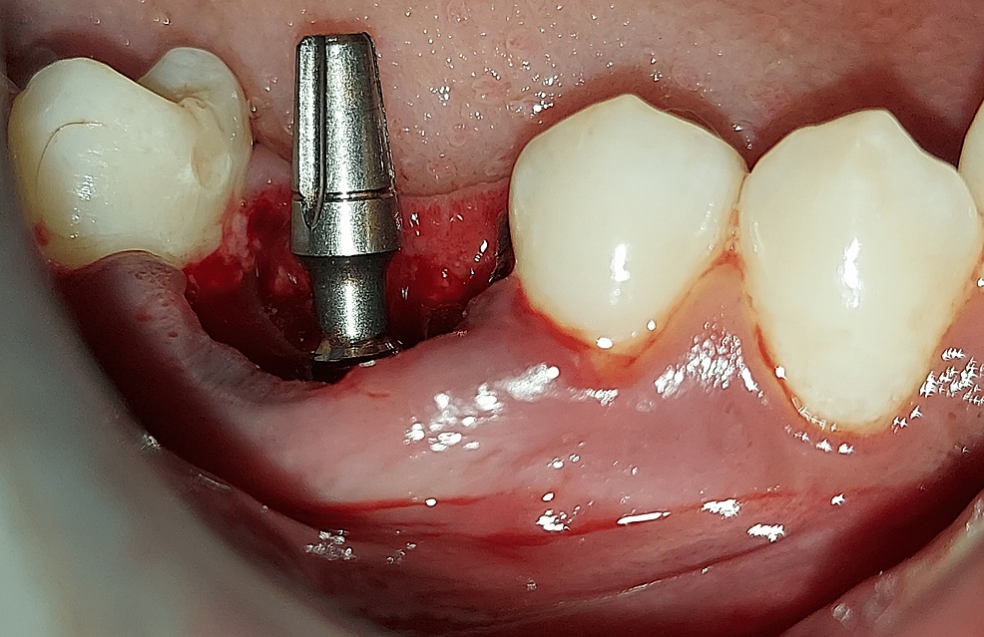
The final implant placement with 46 regions.

Figure 8 depicts the final implant placement done in CO.

**Figure 9:**
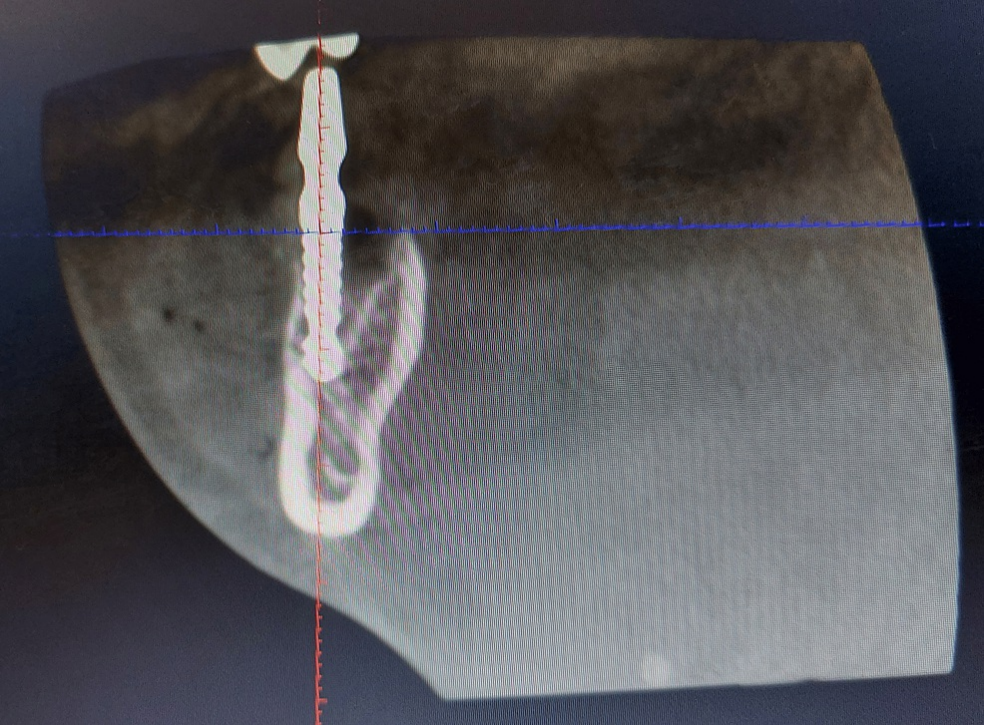
CBCT immediately after implant placement to check the position of the implant. CBCT: cone beam computed tomography.

The implant was adjusted according to angulation by bending the abutment neck. Once the final implant placement was assessed, an immediate postoperative CBCT was taken, as shown in Figure 9.

**Figure 10:**
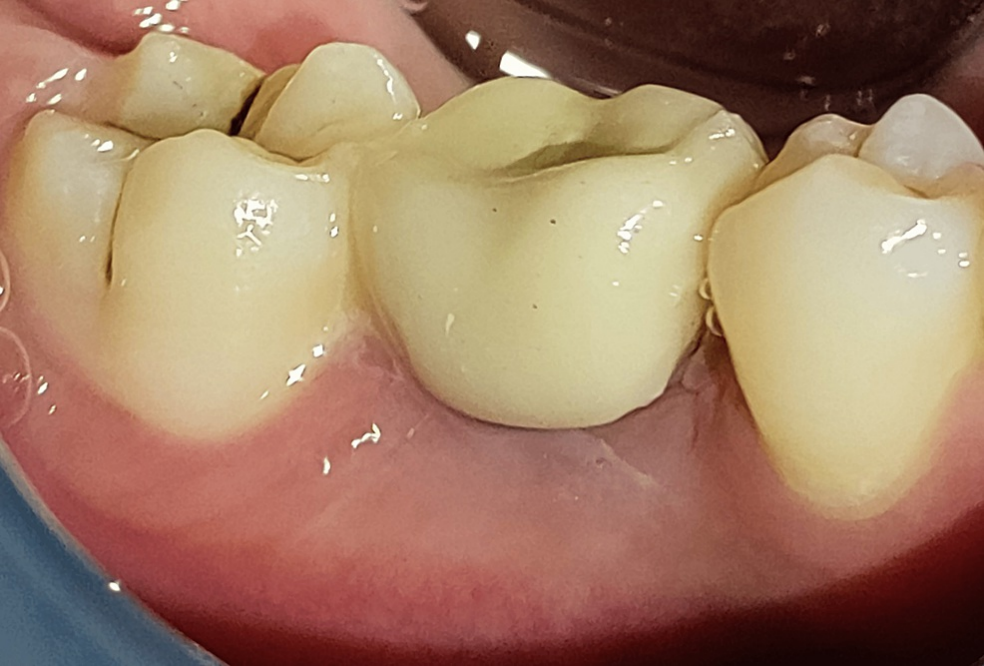
Final prosthesis with 46 regions.

The final prosthesis is in place, as shown in Figure 10.

## Discussion

The basal implant is a recent concept. The various consensus reports on basal implantology have given satisfactory outcomes concerning its placement, immediate loading, and the concept of osseofixation. Since then, multiple studies have been done to accumulate data on its success and survival rates. Although large-scale studies have not been done, reports of cases with a follow-up period of one to five years have significantly demonstrated increased survival rates. Garg et al. [15] investigated the survival of endosseous (immediate loading (IL)) implants and basal IL implants. Fifty-two implants, 34 endosseous and 18 basal, reported satisfactory results. Our case report shows no mobility after crown placement, and the prosthesis achieves proper functional needs.

The patient presented with CO. In this case, we had two options: (1) tooth extraction with the removal of CO lesion followed by GBR using either allograft or autograft and (2) immediate placement of a dental implant. The placement of a conventional implant was not feasible as it would hamper the osseointegration due to the loss of buccal plate and low density of bone. Hence, we decided to place the basal implant (KOC Plus) by osseofixating it into the lesion and allowing osseointegration. The placement of basal implant served numerous advantages. It reduced the duration of therapy and prevented the need for additional procedures.

In their case report, Ghalaut et al. assessed complete mouth rehabilitation in a patient with multiple periodontally compromised teeth. With both maxillary and mandibular cement-retained fixed partial dentures, 18 single-piece basal implants were placed. They can be inserted into the healed bone and the extraction sockets. Their structural qualities enable placement in the bone, which lacks width and height. In cases when (unpredictable) augmentations are a component of an alternative therapeutic strategy, basal implants are the devices of first choice. All issues with conventional (crestal) implantology are resolved by the basal implantology method [16]. When placed in patients with compromised bone or alveolar ridges, immediately loaded basal implants were evaluated clinically, radiographically, and functionally. Anuradha et al. [17] concluded that all implants were successful, with no incidence of infection and no mobility at the end of the six-month study period.

The dental rehabilitation of individuals with cleidocranial dysplasia (CCD) was described by Ahmad et al. in their case study as a case-sensitive process needing a multidisciplinary approach. Skeletal and oral CCD symptoms were evident in a 24-year-old woman. She has several impacted and improperly erupted teeth. Under general anesthesia, all impacted and incompletely erupted teeth were removed, and full dentures were made and delivered. Fourteen basal implants were placed in the maxillary and mandibular jaws three months later and three days after they were immediately loaded with fixed prostheses. As a result, the authors concluded that basal implant-supported prostheses might be helpful in CCD patients who have a reduced bone foundation following tooth extraction. The procedure takes less time than orthodontic therapy, does not involve bone grafting, spares the patient from wearing uncomfortable dentures, lowers total costs, and enhances the quality of life [18].

In their case study, Awadalkreem et al. [19] assessed fixed basal implant-supported prostheses for the prosthetic rehabilitation of maxillary and mandibular gunshot injuries. A 32-year-old individual had been shot in the peripheral mandibular region. A multidisciplinary team developed the following treatment strategy: stage 1 root canal therapy for the anterior maxillary teeth, crown fabrication, and transitional mandibular removable partial denture fabrication. Stage 2 involves the installation of final prostheses that are immediately mounted into six corticobasal screw implants. The patient had good peri-implant soft tissue health, prosthesis stability, and appreciable improvements in appearance and functionality after five years of usage.

Patients’ satisfaction while switching from traditional implant prostheses to basal implant-supported prostheses was assessed by Awadalkreem et al. [20] and reported an improved patient satisfaction score.

This case report presented a novel technique of using the basal implant to its best possible advantage. We believe that clinicians should comply with patients’ requests. Patients avidly seek early outcomes from implant therapies. The mainstream dentistry profession today is working to enhance immediate load implant methods. This is not always feasible with conventional treatment modalities. Although most implantologists do not favor basal implants, the evidence suggests that they are an excellent alternative under most circumstances when it comes to bone augmentations. The philosophy of placement of basal implants differs from conventional implantology, which depends solely on osseofixation rather than mere osseointegration.

## Conclusions

The concept of basal implantology is an innovative and reliable technique for patients demanding immediate rehabilitation of compromised ridges. Basal implants make dental implant procedures accessible, secure, and economical for everyone, including smokers, people with advanced periodontitis, and people with controlled diabetes. Moreover, it eliminates all the drawbacks of conventional implantology. And hence, it is rightly said that sometimes the best solutions are unconventional.
